# Value-Based Care: Implications for nursing

**DOI:** 10.1097/NSG.0000000000000133

**Published:** 2024-01-24

**Authors:** Adele Webb

**Affiliations:** At Strategic Education, Inc. based in Minneapolis, Minn., **Adele Webb** is the Executive Dean of Healthcare Initiatives.

**Keywords:** care coordination, chronic disease management, healthcare delivery model, personalized medicine, population health, value-based care, VBC

## Abstract

The concept of value-based care (VBC) shifts the focus from volume to quality in healthcare. For nursing, this means focusing on patient outcomes, preventive care, and cost efficiency. This article discusses the critical role of nurses in VBC, particularly in coordinating care, improving patient satisfaction, and driving overall healthcare effectiveness and value.

**Figure:**
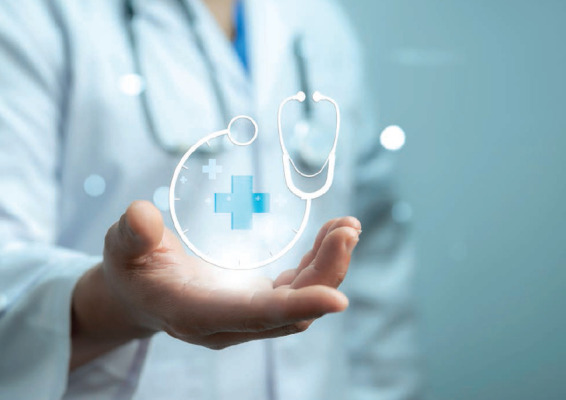
No caption available.

Value-based care (VBC) is a healthcare delivery model in which healthcare organizations, physicians, and advanced practice clinicians (APCs) are compensated based on patient health outcomes rather than the volume of services provided.^1^ The model incentivizes nurses, physicians, and APCs to deliver high-quality care while controlling costs.

VBC was developed as a response to escalating healthcare costs and concerns of poor quality of care.^1^ Envisioned as a component of the Affordable Care Act (ACA), the goal was to address the Institute for Healthcare Improvements Triple Aim that called for enhancing the patient experience, improving patient outcomes, addressing population health and the social determinants of health (SDOH) as well as reducing cost.^2^

The focus on value rather than volume strives to create a sustainable healthcare system that provides patients with higher-quality care while reducing costs. With its comprehensive approach, the VBC model requires clinicians to shift from a fee-for-service focus to prioritizing quality, patient-centered care, cost efficiency, care coordination, and preventive care (see *Key components of VBC*).

**Table:** Key components of VBC

Component	Description
Patient-centered care	Focuses on providing care that is respectful of and responsive to individual patient preferences, needs, and values, ensuring that patient values guide all clinical decisions
Care coordination	Ensures that care is seamless across all healthcare professionals, including primary care, specialists, and other support services, to avoid duplication and improve efficiency
Evidence-based practice	Emphasizes the use of clinical data and research to guide decisions, ensuring treatments are backed by proven effectiveness and best outcomes for the patient
Preventive health	Focuses on preventing diseases before they occur by encouraging wellness visits, vaccinations, screenings, and lifestyle modifications to improve long-term health outcomes
Outcome-based payments	Shifts from traditional fee-for-service models to paying providers based on patient health outcomes, rewarding improved patient health rather than the volume of services provided
Cost efficiency	Encourages the use of the most cost-effective treatments and reduces unnecessary or redundant procedures, tests, and hospitalizations to lower healthcare costs for patients and providers
Patient engagement	Actively involves patients in their care decisions, providing education and tools to help them manage their health, which improves compliance and overall outcomes
Health IT and analytics	Utilizes EHRs and data analytics to track patient outcomes, improve care coordination, and identify areas for improvement in care delivery

A specific example of nurses focusing on value rather than volume can be seen in the management of patients with chronic diseases, such as diabetes. Instead of scheduling frequent appointments for routine checkups, nurses in a VBC model may implement a patient-centered approach by developing individualized care plans that could include regular follow-up via telehealth, remote monitoring of blood glucose levels, and providing education on self-management techniques.

Nurses are well-positioned to avoid gaps in services, reducing poor patient outcomes.^1^ By focusing on improving the patient's overall health and preventing complications, nurses help reduce hospital readmissions and ED visits. This approach emphasizes quality outcomes, care coordination, and long-term health improvements rather than the sheer number of patient interactions, aligning with the goals of VBC.

This article discusses the critical role of nurses in VBC. By implementing this model, nurses can concentrate on evidence-based practice, tailor patient-care plans and care coordination, ensure early intervention, and eliminate waste and unnecessary use of resources.

## Patient-centered care

A major focus of VBC is involving patients and families in care coordination. Research has found that incorporating patient and family input and involvement into a care plan results in increased adherence to the plan and better patient outcomes.^3^ Nurses have the opportunity to achieve this by providing patient education, enabling patients and families to become knowledgeable about their condition. By educating patients and families on the management of chronic diseases, adherence to medication regimens and treatment plans, and adopting a healthy lifestyle, the VBC model supports patients' ability to manage their own care and include their personal preferences in the plan.

## Care coordination and integration

From admission to discharge, nurses often serve as care coordinators who are responsible for managing continuity of care, transitioning between care settings, and identifying and resolving barriers to care.

Improving care coordination is a major concept in the VBC model. Using new and emerging technology, nurses can seamlessly track progress toward goals, collaborate with all roles in the healthcare team, and work with patients and families to help them become active participants in their care. For example, nurses can use telehealth platforms to provide virtual care, allowing them to remotely monitor patients, conduct virtual consultations, and manage chronic conditions. Nurses can track patients' vital signs in real-time through wearable devices like smartwatches or continuous glucose monitors, allowing them to intervene earlier when issues arise. This use of technology enhances care coordination, promotes preventive care, and reduces unnecessary hospital visits.^4^

## Population health management

Population health is an interdisciplinary, customizable approach that allows health departments to connect practice to policy for change to happen locally. Population health works within nontraditional partnerships in the community, such as public health, industry, academia, healthcare, and local government entities, to achieve positive health outcomes.^5^ Population health concentrates on the health status and health outcomes of groups of people. A population is as one defines it: It could be patients with similar diagnoses or with the same physician or APC, people in the same zip code, people in prison, or people in a particular church.

Population health also focuses on the SDOH.^6^ By paying attention to concepts such as level of education, neighborhood safety, access to healthy foods, and socioeconomic status, nurses highlight significant health concerns and address the allocation of resources to improve health conditions for the defined population.

Nurses are in a key position to recognize and address these determinants, as they often have close contact with patients across various settings. For example, consider a nurse working with a patient recovering from surgery who lives in an underserved community. If the patient lacks access to proper nutrition or lives in unsafe housing conditions, these factors could delay healing or lead to health complications. The nurse could coordinate care by involving social workers or community health programs to ensure the patient has access to a safe living environment and proper nutrition.

## Personalized medicine

Personalized medicine (also known as precision medicine) tailors healthcare to one's characteristics, specifically environmental, lifestyle, and genetic information, to offer care that is more likely effective.^7^ Early disease detection and discovering biomarkers that guide treatment are revolutionary ways that personalized medicine is shifting healthcare. Personalized medicine can help prevent ineffective treatments and potential adverse reactions from such treatments and possible disease progression.^7^

Preventive care is the cornerstone of VBC.^5^ For both healthy patients and patients with chronic diseases, prevention can avoid costly treatments and keep patients out of the hospital. The crux of patient-centered care, this model can provide precise risk assessments and targeted prevention and treatment. In addition, patients and families are more likely to be engaged in their care when they understand that the treatment plan is tailored to their situation.

Personalized medicine and VBC have closely aligned approaches that are changing healthcare delivery, with common goals to enhance disease prevention, patient outcomes, and cost efficiency.

## Benefits of VBC

The discussion of VBC components demonstrates the benefits of this model for patients, nurses, and healthcare systems. Other benefits include patient-centered care for a patient managing a chronic illness, for whom it is easy to overlook how the patient is adapting to changing healthcare needs. Consider a patient chronically ill after a kidney transplant. After being discharged with a myriad of orders and restrictions, the patient begins to get frustrated. Without input about how the patient would like to spend recovery time, the patient begins doing the things they believe as important or essential. These things include self-medication, alternative medicine, ED visits, and online research and diagnosis, to name a few. The practice of stretching the limits, often ending up as a failed transplant or hospital readmission, can be averted if the nurse takes the time to listen to how the patient views recovery.

Care coordination provides another opportunity for nurses to ensure quality patient outcomes.^8^ Think about a patient newly diagnosed with cancer. Imagine the patient's angst and fear. When discharged with only a referral to a physician or APC, the waiting can become unbearable. A 2-week lag before an appointment, then a subsequent 2-week wait for an oncology referral can result in increasingly failing health and visits to the ED.

However, examining this situation through a VBC lens, a patient waiting weeks between appointments without proper support contrasts sharply with VBC's focus on population health, which emphasizes care coordination, health equity, interdisciplinary collaboration, and patient engagement. Nurses are essential to effectively implementing VBC by ensuring timely referrals; providing emotional and educational support; and collaborating with other nurses, physicians, and APCs. By doing so, they help bridge the gaps in care, reducing patient anxiety and improving health outcomes.

Implementing a VBC model can also lower costs for both the patient and the organization while raising value.^9^ Tailoring patient care and coordinating medical testing and procedures can help reduce wasteful inefficiencies and health disparities and more effectively steward funds to support care priorities.^5^

As VBC continues to grow across the nation, the overall satisfaction of physicians and APCs is expected to grow.^10^ Better management of chronic diseases will result in healthier patients and fewer unnecessary visits, allowing nurses more time and flexibility.^1^

## Challenges of VBC

Transitioning to VBC is not without challenges. Nurses will need to develop new competencies in areas such as data analysis, quality improvement, and interprofessional collaboration. Electronic health records will need to be adapted to include digital tools. Considering organizational culture, strong leadership will be required to facilitate implementation. A shift to VBC is a cultural shift. Nurses will need to focus more on patient outcomes. This could also result in additional time and resources, stressing an already stressed workforce.

Also, as technological advances explode in healthcare, there will be a need for nurses to implement new artificial intelligence tools and find ways to contribute to innovation. Finally, nurses will need to be empowered to contribute to model implementation and advocate for necessary resources and support.^1^

## Nursing education programs

The concepts of VBC must be integrated into nursing curricula. In 2021, the American Association of Colleges of Nursing published the document *Core Competencies for Professional Nursing Education*^11^ which outlines the necessary competencies for nursing education, many of which align with VBC principles. A focus on holistic patient care, interprofessional collaboration, data-driven practice, preventive and population health, and cost-conscious care are all competencies that could be added to a nursing curriculum. Incorporating these competencies into nursing curricula will contribute to preparing future nurses for a career in VBC.

## Future of VBC

The future of VBC is promising, with numerous advancements in payment models, technology, patient engagement, interdisciplinary collaboration, and policy support driving the transformation.^1^ By continuing to focus on these areas, the healthcare system can achieve the dual goals of improving patient outcomes and reducing costs.

For nurses specifically, the future of VBC includes an expanded scope of practice, greater reliance on technology, and a strong focus on patient engagement and preventive care. Nursing interventions such as patient and family engagement, interdisciplinary collaboration, and clear and effective communication can result in fewer readmissions, better patient outcomes, and lower costs of care.
